# Scleral buckling as a standalone surgery in the era of pars plana vitrectomy: a 10-year retrospective cohort study

**DOI:** 10.1186/s40942-025-00785-z

**Published:** 2025-12-30

**Authors:** Yosra Er-reguyeg, Moncef Berkache, Mélanie Hébert, Georges Hallak, Sihame Doukkali, Eunice Linh You, Serge Bourgault, Mathieu Caissie, Éric Tourville, Ali Dirani

**Affiliations:** 1 Faculty of Medicine, Laval University, Quebec, QC Canada; 2 Department of Ophthalmology, Centre Hospitalier de l’Université de Montréal (CHUM), Montréal, QC Canada; 3 Department of Ophthalmology, Hôpital du Saint-Sacrement, Laval University, 1050 Chemin Sainte-Foy, D2-44C, Québec, QC G1S 4L8 Canada

**Keywords:** Scleral buckling, Rhegmatogenous retinal detachment, Single surgery anatomical success, Vitreoretinal surgery, Retina

## Abstract

**Background:**

Scleral buckling (SB) is an established treatment for rhegmatogenous retinal detachment (RRD). Once the gold standard for RRD repair, SB became less performed with the rise of pars plana vitrectomy (PPV). This retrospective interventional cohort study aims to provide 10 years of real-world data on SB surgical outcomes in a Canadian tertiary eye center.

**Methods:**

Patients undergoing primary SB surgery at the CHU de Québec between January 2014 and December 2023 for primary RRD with at least 3 months of postoperative follow-up were included. Multiple linear and logistic regression models were developed to identify variables associated with final BCVA and single surgery anatomical success (SSAS).

**Results:**

A total of 187 phakic patients undergoing primary SB surgery with a median [Q1, Q3] follow-up (FU) of 23.0 [11.0, 38.9] months were included. Females constituted 56.1% (*n* = 105) of the cohort with a median age at surgery of 36.9 [28.6, 47.0] years. Most patients did not have a preoperative posterior vitreous detachment (*n* = 161, 86.1%). Macula status was on for 109 (58.3%), off for 46 (24.6%), and split for 32 (17.1%) patients. Median RRD extent was of 4.0 [3.0, 5.0] clock hours, with the preoperative number of retinal breaks being of 2.0 [1.0, 3.0]. Inferior RRD associated with inferior breaks was present in 101 (54.0%) eyes. SSAS was achieved in 169 (90.4%) cases, with 184 (98.4%) patients achieving retinal reattachment at last follow-up. BCVA progressed from baseline median values of 0.14 [0.00, 0.40] logMAR, improving by 12 months (0.12 [0.02, 0.30], *p* = 0.004) and by final follow-up (0.10 [0.00, 0.28], *p* < 0.001). Male sex was a negative predictor of SSAS (*p* = 0.008), while SF_6_ gas tamponade was a positive predictor (*p* = 0.035). Chronicity of RRD (*p* = 0.012) and worse preoperative BCVA (*p* < 0.001) were associated with worse final BCVA.

**Conclusions:**

The use of primary SB as a standalone surgery continues to be a safe and effective technique for RRD repair, specifically in young phakic patients with no PVD; or when PPV may not be the preferred option to preserve lens state and avoid iatrogenic retinal breaks, two well-documented complications of vitrectomy.

## Background

Rhegmatogenous retinal detachment (RRD) is an ophthalmic emergency characterized by separation of the neurosensory retina from the retinal pigment epithelium (RPE) due to a retinal break that allows liquefied vitreous to enter the subretinal space [1, 2]. The annual incidence of RRD is roughly 10–18 per 100,000 people [2], with higher rates in older adults and myopic individuals.

The first widely used surgical treatment for RRD repair was the scleral buckle (SB). This procedure, introduced in the 1940s by Ernst Custodis and popularized by Charles Schepens in the 1950s, quickly became standard of care. SB involves indenting the scleral wall usually with a silicone band or sponge to relieve vitreoretinal traction and permanently close retinal breaks, often combined with cryotherapy to create a chorioretinal adhesion. Over the ensuing decades, refinements in SB technique such as drainage of subretinal fluid and encircling elements led to higher reattachment rates: modern series report primary success in approximately 85–93% of cases [3, 4].

Pars plana vitrectomy (PPV) emerged in the 1970s and became increasingly popular in the 1990s and 2000s as vitrectomy technology advanced [5]. Innovations such as wide-angle viewing systems and small-gauge instrumentation have made PPV more efficient and accessible, contributing to a significant shift in RRD repair trends [6, 7]. In contemporary practice, SB is performed in less than 20% of primary RRD repairs [8]. The decline in SB utilization is often attributed not to inferior outcomes, but to changes in surgical training and comfort as many younger retinal surgeons are less experienced with the technical nuances of buckling [9].

Recent studies confirm that results from SB are at least equivalent to PPV in many indications and can be most appropriate in specific cases [10, 11]. Interest thus remains in the “forgotten art” of scleral buckling as a valuable tool in the modern management of RRD.

Given these evolving paradigms, it is essential to re-evaluate the performance of scleral buckling using contemporary data. This study aims to assess the anatomic and functional outcomes of SB for primary RRD repair over a 10-year period at a Canadian tertiary center, and to identify factors predictive of surgical success.

## Methods

### Study design and population

This retrospective single center interventional cohort study included 187 patients who underwent primary scleral buckling surgery between January 2014 and December 2023 for RRD at the Centre Universitaire d’Ophtalmologie – Centre Hospitalier Universitaire de Québec (CUO–CHU de Québec) with minimally 3 months of postoperative follow-up. This study was done in accordance with the tenets of the Declaration of Helsinki and received prior Institutional Review Board (IRB) approval from the same institution. Patients with non-rhegmatogenous RD, as well as those undergoing SB as secondary surgery were excluded. We excluded non-idiopathic cases, i.e., eyes with a history of retinopathy of prematurity, endophthalmitis, uveitis or other inflammatory/infectious eye condition, age-related macular degeneration, and proliferative diabetic retinopathy.

### Outcome measures and definitions

The primary anatomical outcome measure was single-surgery anatomical success (SSAS), defined as the absence of RD recurrence requiring an additional surgical intervention at any time during follow-up, irrespective of whether reoperation was ultimately performed. Cases requiring additional cryotherapy or laser or pneumatic retinopexy to barricade fluid or new breaks were also considered as SSAS, while eyes which required vitrectomy with gas or silicone tamponade were classified as failures. Surgical failure was not considered a postoperative complication. The primary functional outcome measure was final postoperative best-corrected visual acuity (BCVA), measured in logarithm of the minimum angle of resolution (logMAR). Patients were considered to have high myopia if their refractive error was equal or exceeded 6 diopters (D). Macula status was documented as on, off, or split. Incidence and grade of proliferative vitreoretinopathy (PVR) was documented using the 1983 Retina Society classification [13]. Chronicity of RRD was also assessed, with chronic RRD being defined as lasting 14 days or more from symptom onset. Retinal breaks were characterized by number, extension (number of clock hours), and type. Inferior RRD was defined as retinal detachment with breaks located between 4:00 and 8:00. If preoperative findings differed from intraoperative findings, intraoperative data was collected as deemed more accurate. As means to quantify the severity of postoperative complications and assess surgical safety, we classified all adverse events using the Classification of Ophthalmological Complications (COC) grading system [14]. Given prior evidence suggesting that RRD presents with distinct phenotypical features across under 40 and over 50 age groups, a subgroup analysis stratified by age (< 40 vs. ≥ 40 years) was conducted [15].

### Surgical procedure

Surgery was performed by one of five fellowship-trained vitreoretinal surgeons. Briefly, after retrobulbar and/or general anesthesia and akinesia, a 360-degree conjunctival peritomy was performed, followed by the placement of traction sutures on the recti muscles to stabilize the eyeball. Indirect ophthalmoscopy was conducted to localize and mark retinal breaks, which were then sealed using cryotherapy. Circumferential buckle elements were placed on the sclera utilizing scleral tunnels or secured with sutures. Drainage of subretinal fluid (SRF) and addition of gas tamponade (i.e., air, sulfur hexafluoride (SF_6_) or perfluoropropane (C_3_F_8_)) were done at the discretion of the surgeon.

### Statistical analysis

Data were analyzed using IBM SPSS Statistics (IBM, Armonk, NY, USA) and statistical significance was set at a p-value of < 0.05. Normally distributed continuous variables were presented as means ± standard deviations, while non-normally distributed continuous variables were presented as medians with interquartile ranges [first quartile (Q1), third quartile (Q3)]. Categorical variables were presented as frequencies (percentages). Variables were compared using independent Student’s t-tests for normally distributed continuous variables, Mann-Whitney U tests for non-normally distributed continuous variables, Pearson chi-square tests for categorical variables, and Wilcoxon signed-rank tests for non-normally distributed paired preoperative and postoperative continuous variables. A logistic regression model was developed to assess variables associated with SSAS. It incorporated the following baseline and perioperative characteristics: sex, follow-up period, age at surgery, type of detachment, presence of RRD with inferior breaks, chronicity of RRD, presence of PVR preoperatively, presence of preoperative vitreous hemorrhage or opacity, lattice degeneration, tamponade, and SRF drainage. Variables were included based on the criterion of at least 5–10 cases of RRD recurrences per variable in the final model [16]. The logistic regression model was built using backward elimination with a p-value removal criterion of 0.2. A multiple linear regression model was built to study associations with final BCVA, incorporating the same variables as those used in the logistic regression model, along with SSAS and baseline BCVA as additional inputs. The model was built using backward elimination with an F-to-remove threshold of 0.2. Unstandardized coefficients with 95% confidence intervals (CI) and standardized coefficients were produced.

## Results

### Demographics

This study included 187 phakic eyes of 187 patients with 56.1% (*n* = 105) being female and a median age at surgery of 36.9 [28.6, 47.0] years. The cohort consisted of 80 (42.8%) cases being 40 years old or older, and 107 (57.2%) cases being younger. The median follow-up period was of 23.0 [11.0, 38.9] months. Preoperative posterior vitreous detachment (PVD) was present in 26 (13.9%) cases. Further characteristics of the studied population are presented in Table 1.

**Table 1:** Preoperative characteristics of studied population

Variable		*n* = 187
Age at surgery (years), median [Q1, Q3]		36.9 [28.6, 47.0]
Female sex, n (%)		105 (56.1)
Follow-up period (months), median [Q1, Q3]		23 [11.0, 38.9]
Spherical equivalent (D), median [Q1, Q3]		-5.0 [-7.0, -1.5]
Presence of high myopia (≥ 6D), n (%)		65/164 (39.6)
Presence of lattice degeneration, n (%)		78 (41.7)
Presence of PVD, n (%)		26 (13.9)
Partial		14 (7.5)
Complete		12 (6.4)
Preoperative PVR Grade, n (%)*		
A		2 (1.1)
B		6 (3.2)
C1		11 (5.9)
C2		6 (3.2)
C3		0 (0.0)
Vitreous hemorrhage/opacity, n (%)		22/186 (11.8)

D, diopter; PVD, posterior vitreous detachment; PVR, proliferative vitreoretinopathy

*: Preoperative PVR grade was not available for two patients

The median time between diagnosis of RRD and surgery was of 1.0 [1.0, 5.0] day, while the median time between the onset of symptoms and surgery was 14.0 [5.3, 34.5] days. RRD was chronic in 93/173 (53.8%) of cases. Regarding RRD characteristics, median RRD extent was of 4.0 [3.0, 5.0] clock hours and a median of 2.0 [1.0, 3.0] retinal breaks were observed. The macula status was *on* in 109 (58.3%) cases, *off* in 46 (24.6%) cases, and *split* in 32 (17.1%) cases. The retinal breaks causing detachment were predominantly round holes, observed in 154 cases (82.4%), followed by horseshoe tears in 18 cases (9.6%), retinal dialysis in 10 cases (5.3%), retinoschisis in 5 cases (2.7%), and giant retinal tears in 4 cases (2.1%). Inferior RRD associated with inferior breaks was present in 101 (54.0%) eyes. Retinal break type was non identifiable in 3 (1.6%) cases.

In terms of surgical modalities, a circumferential buckle was used in all 187 (100.0%) cases. An encircling element was used exclusively in 184 (98.3%) cases, whereas a segmental element (i.e., #286 tire + #240 band) was added in 3 (1.6%) cases. Otherwise, for the cases for which this information was available, a #41 band was used in 119/167 (71.3%) eyes and a #42 band was used in 48/167 (28.7%) eyes. Chorioretinal adhesion was achieved using cryotherapy for all cases. SRF was drained in 159 (85.0%) cases and anterior chamber (AC) paracentesis was performed for 127 eyes (67.9%) to help manage intraocular pressure (IOP). Adjuvant gas tamponade was added in the form of air in one (0.5%) case, SF_6_ in 102 (54.5%) cases and C_3_F_8_ in 4 (2.1%) cases.

### Anatomic outcomes

SSAS was achieved in 169 (90.4%) cases, while reoperation by PPV was done once for 15 (8.0%) cases, twice for one (0.5%) case and exceptionally 6 times for one other (0.5%) case. Otherwise, 50 (26.7%) patients underwent supplemental laser photocoagulation, 1 (0.5%) patient had cryotherapy and 2 (1.1%) had pneumatic retinopexy postoperatively. By the end of the follow-up period, the retina was successfully attached in 184 cases (98.4%). Logistic regression identified male sex as a negative predictor of SSAS (*p* = 0.008) and SF_6_ gas tamponade as positive predictor (*p* = 0.035) (Table 2**—** Logistic Regression Model for Single Surgery Anatomical Success Following Scleral Buckling Based on Baseline and Perioperative Characteristics).

**Table 2:** Logistic regression model for single surgery anatomical success based on baseline and perioperative characteristics

Characteristic	B	S.E.	OR	95% CI	*p*-value
Male sex	-1.823	0.684	0.161	0.042, 0.617	**0.008**
SRF drainage	1.150	0.650	3.158	0.883, 11.286	0.077
SF_6_ tamponade	1.322	0.628	3.752	1.095, 12.854	**0.035**

Bold values were considered statistically significant

B, log odds coefficient; CI, confidence interval; OR, odds ratio; SE, spherical equivalent; SF_6_, sulfur hexafluoride; SRF, subretinal fluid

### Functional outcomes

**Fig. 1:**
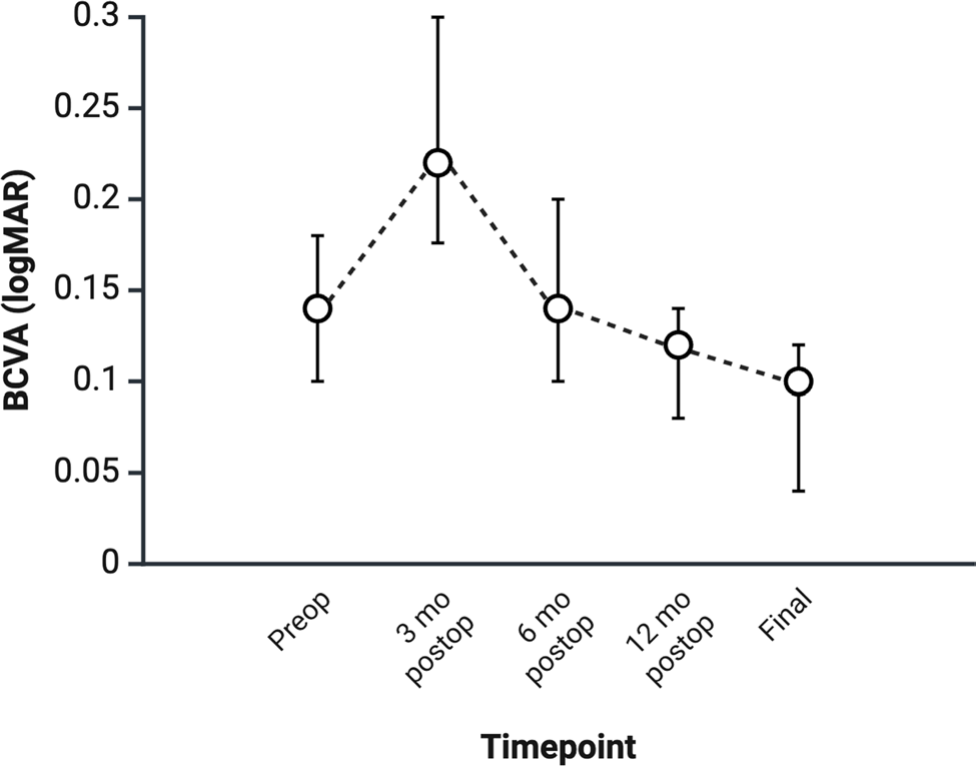
Change in Best-Corrected Visual Acuity (logMAR) from Preoperative to Final Postoperative Timepoint. BCVA improved from a preoperative median of 0.14 [0.00, 0.40] to 0.22 [0.10, 0.44] logMAR at 3 months postoperatively (*p* = 0.355), 0.14 [0.02, 0.40] logMAR at 6 months (*p* = 0.296), 0.12 [0.02, 0.30] logMAR at 12 months (*p* = 0.004), and 0.10 [0.00, 0.28] logMAR at final follow-up (*p* < 0.001). Significant improvements in BCVA were observed between the preoperative period and postoperative 12 months (*p* = 0.004), as well as between 3 and 6 postoperative months (*p* < 0.001), postoperative 3 and 12 months (*p* < 0.001), postoperative 3 months and final follow-up (*p* < 0.001), postoperative 6 and 12 months (*p* = 0.018), postoperative 6 months and final follow-up (*p* < 0.001), and between postoperative 12 months and final follow-up (*p* = 0.013) Graphical data were presented using median ± 95% confidence intervals BCVA, best-corrected visual acuity; logMAR, logarithm of the minimum angle of resolution; preop, preoperative; postop, postoperative

BCVA improved from a preoperative median of 0.14 [0.00, 0.40] to 0.22 [0.10, 0.44] logMAR at 3 postoperative months (*p* = 0.355), 0.14 [0.02, 0.40] logMAR at 6 months (*p* = 0.296), 0.12 [0.02, 0.30] logMAR at 12 months (*p* = 0.004), and 0.10 [0.00, 0.28] logMAR at final follow-up (*p* < 0.001). Figure 1 illustrates the evolution of BCVA over the follow-up period. A multiple linear regression model identified chronic RRD (*p* = 0.012) and lower preoperative BCVA (*p* < 0.001) as parameters significantly associated with poorer final BCVA (Table 3**—** Multiple Linear Regression Model for Final Best-Corrected Visual Acuity Following Scleral Buckling Based on Baseline and Perioperative Characteristics).

**Table 3:** Multiple linear regression model for final best-corrected visual acuity based on baseline and perioperative characteristics

Characteristic	B	95% CI	β	*p*-value
Chronic RRD	0.121	0.027, 0.215	0.160	**0.012**
Preoperative vitreous hemorrhage/opacity	-0.106	-0.251, 0.040	-0.090	0.155
Preoperative BCVA	0.375	0.296, 0.455	0.591	**< 0.001**

Bold values were considered statistically significant

B, unstandardized coefficients; β, standardized coefficients; BCVA, best-corrected visual acuity; RRD, rhegmatogenous retinal detachment

### Postoperative complications

After scleral buckling, a myopic shift was observed, as the SE changed significantly from a median of -5.0 [-7.0, -1.5] D to -7.5 [-10.0, -5.0] D postoperatively (*p* < 0.001). Surgery also resulted in cataract formation, as 14 (7.5%) patients became pseudophakic and one (0.5%) patient became aphakic after surgery. Seven out of these 14 pseudophakic patients and the aphakic patient underwent subsequent PPV for persistent RRD and developed cataract after the secondary intervention. Epiretinal membrane (ERM) and cystoid macular edema/subretinal fluid developed postoperatively in 15 (8.0%) and 10 (5.3%) cases respectively. Intraoperatively, iatrogenic breaks did not occur, while scleral perforation and retinal incarceration was observed in one (0.5%) case. Postoperative hemorrhaging, whether vitreous, subretinal or subchoroidal occurred in 31/184 (16.8%) cases while anterior segment ischemia happened in one (0.5%) case. One patient (0.5%) experienced buckle extrusion, another had suture exposure (0.5%), and buckle infection (0.5%) occurred in a third patient. Postoperative diplopia was reported in 10 (5.3%) patients. At last follow-up, 28 (15.0%) eyes had persistent SRF. Based on the COC grading system, 46 patients (24.6%) experienced no complications, while the remainder exhibited complications ranging from Grade I to V: 45 (24.1%) with Grade I, 32 (17.1%) with Grade II, 38 (20.3%) with Grade III, 24 (12.8%) with Grade IV, and 2 (1.1%) with Grade V.

### Subgroup analysis: under versus over 40 years old

Table 4 depicts the demographic and RRD characteristics of the age-based subgroup analysis. Patients aged ≥ 40 years were more likely to present with preoperative PVD (*n* = 18, 22.5% vs. *n* = 8, 7.5%, p *=* 0.005), and had a higher prevalence of postoperative ERM (*n* = 11, 13.8% vs. *n* = 4, 3.8%, p *=* 0.015). They also exhibited a greater frequency of pseudophakia postoperatively (*n* = 12, 15.0% vs. *n* = 2, 1.9*%*, p *=* 0.001). On the other hand, patients under 40 exhibited more frequently round retinal holes as the causative break type (*n* = 93, 88.6% vs. *n* = 61, 76.3%, *p* = 0.026).

**Table 4:** Characteristics of patients under and over 40 years undergoing primary scleral buckling

Characteristic	< 40 years old *n* = 107	≥ 40 years old *n* = 80	*p*-value
Symptom duration (days), median [Q1, Q3]	15.0 [7.0, 38.8]	10.0 [5.0, 25.0]	0.198
Presence of lattice degeneration, n (%)	46 (43.0)	32 (40.0)	0.398
Preoperative high myopia, n (%)	42/95 (44.2)	23/69 (33.3)	0.196
Preoperative PVD, n (%)	8 (7.5)	18 (22.5)	**0.005**
Presence of preoperative PVR, n (%)	16 (15.2)	11 (13.8)	0.837
Preoperative BCVA (logMAR), median [Q1, Q3]	0.22 [0.02, 0.70]	0.14 [0.00, 0.32]	0.078
Break type: round hole, n (%)	93 (88.6)	61 (76.3)	**0.026**
Final BCVA (logMAR), median [Q1, Q3]	0.10 [0.00, 0.35]	0.10 [0.02, 0.22]	0.910
SSAS, n (%)	97 (92.4)	72 (90.0)	1.000
Attached retina at last FU, n (%)	104 (99.0)	80 (100.0)	0.262
SRF drainage	94 (89.5)	65 (81.3)	0.221
Postoperative lens status, n (%)			
Pseudophakic	2 (1.9)	12 (15.0)	**0.001**
Phakic	104 (99.0)	68 (85.0)	
Aphakic	1 (1.0)	0 (0.0)	
Postoperative myopisation, n (%)	30/39 (76.9)	24/30 (80.0)	1.000
Postoperative PVR worsening/development, n (%)	18 (17.1)	9 (11.3)	0.303
Postoperative diplopia, n (%)	5 (4.8)	5 (6.3)	0.747
Postoperative ERM, n (%)	4 (3.8)	11 (13.8)	**0.015**

Bold values were considered statistically significant

BCVA, best-corrected visual acuity; ERM, epiretinal membrane; FU, follow-up; PVD, posterior vitreous detachment; PVR, proliferative vitreoretinopathy; SSAS, single surgery anatomical success; SRF, subretinal fluid

## Discussion

In our cohort of 187 eyes, SSAS was achieved in 169 (90.4%) cases, rising to 184 cases (98.4%) after additional interventions. These outcomes are in line with the best results reported for both scleral buckling and vitrectomy in modern series. A systematic review and meta-analysis has found primary reattachment rates on the order of 85% to 95% for both SB and PPV, with final success after reoperation reaching approximatively 97% to 99% [17]. A propensity score-matched analysis on phakic eyes with noncomplex RRD found that the risk of surgical failure was significantly higher with PPV compared to SB, reinforcing the anatomical reliability of SB in appropriately selected phakic patients [18]. We identified adjuvant gas tamponade as a significant positive predictor of SSAS, consistent with studies highlighting that gas can enhance retinal reattachment, especially in larger RRDs or eyes with vitreous alterations such as PVD [1]. It is conceivable that these patients underwent more extensive subretinal fluid drainage intraoperatively, leaving the retina almost reattached by the end of surgery. In this context, the gas bubble primarily serves to maintain adequate pressure on the retina, even in patients in whom a PVD may be present. Male sex was a negative predictor of SSAS, possibly due to sex-related differences in vitreoretinal dynamics: men have lower rates of complete PVD and a higher prevalence of persistent vitreoretinal adhesion [19, 20].

A modest but statistically significant visual improvement following SB was observed (0.14 [0.00, 0.40] logMAR preoperatively to 0.10 [0.00, 0.28] logMAR postoperatively, *p* < 0.001), supporting the visual efficacy of SB in this population. Multiple studies and meta-analyses suggest that SB offers superior final BCVA compared to PPV, likely due to cataract formation in phakic eyes after vitrectomy [21]. Preoperative BCVA and the chronicity of retinal detachment were significant predictors of final BCVA in our cohort. Longer symptom duration has been shown to correlate with irreversible photoreceptor damage and poorer visual recovery, even after successful reattachment [22]. Moreover, systematic review results found that preoperative BCVA was the most consistent predictor of final visual outcome across 14 out of 19 included studies on RRD repair [23]. Similarly, a retrospective analysis of traumatic RD showed that baseline BCVA alone explained 55% of the variance in final BCVA [24]. These results reinforce the importance of early intervention and the prognostic value of baseline visual status. The linear regression model also retained preoperative vitreous hemorrhage/opacity in its final iteration, which might seem counterintuitive clinically. However, this variable was not statistically significant (*p* = 0.155), indicating that the negative coefficient likely represents a statistical artifact rather than a true effect. Indeed, the small number of vitreous hemorrhage cases included makes this estimate susceptible to instability, and five of these eyes had markedly poor preoperative vision due to media opacity (1.3–2.6 logMAR) that improved once the hemorrhage cleared, which can disproportionately influence the model.

Being an extraocular surgery, SB avoids many of the intraocular risks associated with vitrectomy, but it has its own spectrum of possible complications. An induced median myopic shift of -2.5 D in buckled eyes was observed, comparable to the axial elongation of approximately 1.0 mm found in similar studies [12]. Persistent SRF was observed in 15% of cases (*n* = 28). Indeed, SRF may persist for a few months despite the closure of all the breaks in some patients. Such eyes can be observed safely as most residual fluid reabsorbs spontaneously over weeks to months without affecting final anatomical outcomes and long-term visual prognosis. Perhaps one of the most feared complications in RRD surgery is PVR. By final follow-up, some cases had newly developed PVR, while others showed progression. This might reflect our cohort’s large fraction of chronic RRDs, which are more prone to PVR [25]. It remains that, with careful patient selection, SB does not inherently carry a higher risk of PVR compared to PPV as the risk is more related to detachment duration, extent, and patient factors [26].

Patients aged ≥ 40 were significantly more likely to present with preoperative PVD and develop postoperative ERM [27]. Since PVD can induce retinal traction leading to horseshoe tears, its higher prevalence in older patients may explain their lower rate of round holes. Conversely, patients under 40 more commonly exhibited round atrophic holes, consistent with typical RRD presentations in phakic eyes with lattice degeneration, and an absence of spontaneous PVD [28, 29].

Utilizing the COC framework allowed standardized, severity-based reporting of surgical outcomes and allowed for comparability to future studies using consistent complication grading. With 115 out of 141 (81.6%) complication cases being able to be managed in an ambulatory setting (i.e., less than grade IV), scleral buckling reflected a favorable safety profile.

Due to reduced emphasis on SB teaching and training in modern vitreoretinal fellowships, a decline in its use has been observed [30]. New techniques have thus been introduced to improve the consistency and safety of scleral buckle placement, including a method using scleral tunnels and precise band shortening to achieve a reproducible 1 mm approximative buckle height [31]. Other new modalities include the chandelier-assisted SB, which incorporates intraocular illumination and wide-angle viewing systems [32, 33]. Newer SRF drainage techniques like guarded needle drainage have also been developed to minimize bleeding risk by limiting trauma to choroidal vessels as well as reducing the risk of retinal incarceration [34].

This study provides a comprehensive 10-year analysis of scleral buckling outcomes in a large, well-defined cohort of phakic patients treated at a Canadian tertiary center, reflecting real-world surgical practice across various surgeons. The detailed documentation of surgical techniques, complication grading using the standardized COC framework, and multivariable regression analyses for both anatomical and functional outcomes contribute to the study’s methodological robustness. Additionally, the stratified subgroup analysis by age enhances the understanding of phenotype-related differences in RRD presentation and outcomes. Limitations of this study include its retrospective nature, which introduces inherent risks of selection bias and information bias, although we adjusted for possible confounders in our regression analyses. The surgery was performed by multiple surgeons, which might induce heterogeneity such as variability in technique and postoperative management and thus influence outcomes. On another hand, the fact that it is a single-center study limits its generalizability to other practice settings with different patient populations or surgical preferences. Finally, the absence of a contemporary control group treated with PPV limits direct comparative interpretation, though relevant literature was used to contextualize the findings. As a result, interpretations regarding the relative effectiveness of SB must rely on indirect comparisons with published PPV outcomes, which vary across studies in terms of case selection, techniques, and reporting standards.

The use of primary SB as a standalone surgery continues to be a safe and effective technique for RRD repair, specifically in young phakic patients with no PVD; or when PPV may not be the preferred option to preserve lens state and avoid iatrogenic retinal breaks, two well-documented complications of vitrectomy.

## Data Availability

The datasets used and analysed in the current study are available from the corresponding author on reasonable request.
